# Clinical Characteristics and Long-Term Outcomes of Patients With Fibrosing Mediastinitis: A Multicenter Retrospective Cohort Study

**DOI:** 10.1016/j.jacasi.2026.04.022

**Published:** 2026-06-12

**Authors:** Hongling Su, Juan Wang, Suqiao Yang, Lan Wang, Sugang Gong, Wei Ma, Yiwei Shi, Cheng Hong, Mengyu Cheng, Yunhui Zhang, Xin Pan, Xin Jiang, Bowen Jin, Fajiu Li, Kedong Zhang, Faming Jiang, Wei Huang, Peng Jiang, Jingyun Yang, Peijing Yan, Guo Wei, Yuanhua Yang, Zhicheng Jing, Yunshan Cao

**Affiliations:** a Department of Cardiology, Pulmonary Vascular Disease Center (PVDC), Gansu Provincial Hospital, Lanzhou, China; b Department of Cardiology, General Hospital of Ningxia Medical University, Yinchuan, China; c Department of Respiratory and Critical Care Medicine, Beijing Chao-Yang Hospital, Capital Medical University, Beijing, China; d Department of Cardio-Pulmonary Circulation, Shanghai Pulmonary Hospital, Tongji University, Shanghai, China; e Department of Cardiology, Peking University First Hospital, Peking University, Beijing, China; f Shanxi Province Key Laboratory of Respiratory, Department of Pulmonary and Critical Care Medicine, The First Hospital of Shanxi Medical University, Taiyuan, China; g State Key Laboratory of Respiratory Disease, National Clinical Research Center for Respiratory Disease, Guangzhou Institute of Respiratory Health, The First Affiliated Hospital of Guangzhou Medical University, Guangzhou, China; h Department of Respiratory and Critical Care Medicine, Shanxi Bethune Hospital, Shanxi Academy of Medical Sciences, Tongji Shanxi Hospital, Third Hospital of Shanxi Medical University, Taiyuan, China; i Department of Respiratory and Critical Care Medicine, The First People's Hospital of Yunnan Province, Kunming, China; j Department of Cardiology, Shanghai Chest Hospital, Shanghai Jiao Tong University, Shanghai, China; k Department of Cardiology, Guangdong Cardiovascular Institute, Guangdong Provincial People's Hospital, Guangdong Academy of Medical Sciences Southern Medical University, Guangzhou, China; l Congenital Heart Disease Center, Wuhan Asia Heart hospital, Wuhan, China; m Department of Respiratory and Critical Care Medicine, Affiliated Hospital of Jianghan University, Wuhan, China; n Department of Respiratory and Critical Care Medicine, General Hospital of Ningxia Medical University, Yinchuan, China; o Department of Respiratory and Critical Care Medicine, West China Hospital, Sichuan University, Chengdu, China; p Department of Cardiology, The First Affiliated Hospital of Chongqing Medical University, Chongqing Medical University, Chongqing, China; q Department of Respiratory, General Hospital of Xinjiang Military Region, Urumqi, China; r Rush Alzheimer's Disease Center, Department of Neurological Sciences, Rush University Medical Center, Chicago, Illinois, USA; s Clinical Research Center, Sichuan Provincial People's Hospital, University of Electronic Science and Technology of China, Chengdu, Sichuan, China; t Division of Epidemiology, Department of Internal Medicine, University of Utah School of Medicine, Salt Lake City, Utah, USA; u Heart, Lung and Vessels Center, Sichuan Provincial People's Hospital, University of Electronic Science and Technology of China, Chengdu, Sichuan, China

**Keywords:** fibrosing mediastinitis, prognosis, pulmonary hypertension, tuberculosis

Fibrosing mediastinitis (FM) is a rare disorder characterized by excessive fibrous tissue proliferation replacing normal mediastinal fat, leading to compression or invasion of critical mediastinal structures including pulmonary veins, pulmonary arteries and bronchi.^1^^,^^2^ These pathological changes may result in pulmonary hypertension (PH), right heart failure, and death. The etiology of FM remains unclear. Although tuberculosis (TB) has been proposed as a trigger in China,^3^^,^^4^ robust evidence is lacking, and long-term survival data and prognostic factors remain poorly defined.

We conducted a multicenter retrospective cohort study (ChiCTR2000037899) including 369 FM patients from 16 referral centers in 10 provinces in China between May 2010 and September 2021. The study was approved by the Ethics Committee of Gansu Provincial Hospital with a waiver of informed consent. FM was diagnosed based on contrast-enhanced chest computed tomography showing fibroproliferative soft tissue with loss of normal fat planes, with or without calcification, and compression of adjacent structures. Additional imaging features included atelectasis, mosaic perfusion, ground-glass opacities, interlobular septal thickening, and systemic collateral vessels.^5^^,^^6^ Diagnosis was confirmed by consensus of experienced clinicians and radiologists. Collected data included demographics, symptoms, comorbidities, TB history, N-terminal pro–B-type natriuretic peptide (NT-proBNP), arterial blood gas results, imaging findings, World Health Organization functional class (WHO-FC), and echocardiographic parameters such as tricuspid regurgitation velocity, estimated pulmonary arterial systolic pressure (ePASP), and right ventricular inner diameter.^7^ Suspected PH was defined as an ePASP >40 mm Hg. The primary endpoint was all-cause mortality. Follow-up was completed through April 2, 2022.

Survival was estimated using Kaplan-Meier analysis. Cox regression was applied to identify independent prognostic factors. Variables significant in univariate analysis (*P* < 0.05) were entered into multivariable models. Missing data were imputed using the MissForest algorithm.

The mean age at diagnosis was 65.1 ± 11.0 years, with a mean diagnostic delay of 4.5 ± 5.0 years. Women accounted for 57.5% of patients (Figure 1A). A history of TB was present in 46.6% and 45.3% was classified as idiopathic. The most common symptoms were dyspnea (96.5%) and cough (65.1%), followed by hemoptysis (15.5%) and lower-limb edema (13.8%). Pleural effusion and atelectasis were observed in 39.3% and 51.2% of patients, respectively. Median NT-proBNP was 309.7 pg/mL. Most patients were classified as WHO-FC II or III. Among 301 patients with available echocardiographic data, 256 (85.0%) were suspected of having PH with a median ePASP of 64.9 mm Hg (Figure 1A). The FM dyad (PH plus atelectasis) was present in 38.3% of cases, and the FM triad (dyad plus pleural effusion) in 18.3%.

**Figure 1:**
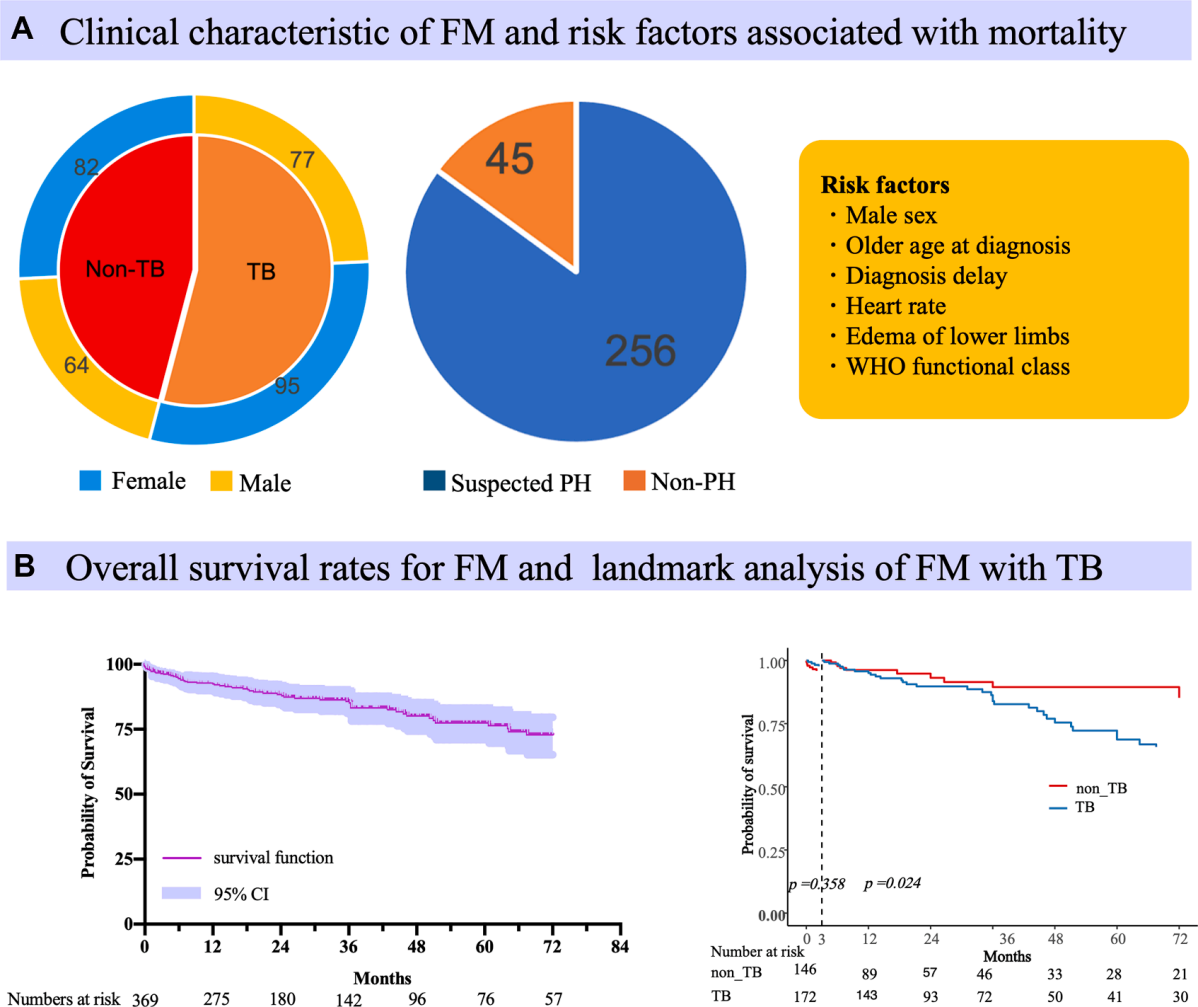
Long-Term Survival and Risk Factors for All-Cause Mortality in FM (A) Clinical characteristics and risk factors associated with all-cause death of FM. (B) Overall 1-, 2-, and 3-year survival rates and landmark survival analysis in patients with a history of TB at 3 months (*P*  =  0.024). FM = fibrosing mediastinitis; PH = pulmonary hypertension; non-PH, defined as estimated pulmonary artery systolic pressure <40 mm Hg; TB = tuberculosis.

Median follow-up was 23.3 months. During follow-up, 66 patients died and 44 were lost to follow-up. The 1-, 2-, and 3-year survival rates were 93.1%, 88.7%, and 86.0%, respectively (Figure 1B). In univariate analysis, mortality was associated with older age, diagnostic delay, male sex, higher heart rate, lower-limb edema, pulmonary embolism, pleural effusion, atelectasis, higher NT-proBNP, worse WHO-FC, and larger right ventricular inner diameter. Multivariate Cox analysis identified male sex (HR: 2.38), older age at diagnosis (HR: 1.04 per year), longer diagnostic delay (HR: 1.04, 95% per year), higher heart rate (HR: 1.02 per beats/min), presence of lower-limb edema (HR: 2.87), and higher WHO-FC (HR: 2.91) as independently predictors of mortality (Figure 1A).

Landmark survival analysis showed that beyond 3 months after diagnosis, patients with a TB history had significantly worse survival than those without TB (Figure 1B).

FM is frequently misdiagnosed because of its nonspecific manifestations, with an average diagnostic delay of nearly 5 years. Nearly half of patients exhibited atelectasis, which may serve as an important imaging clue for earlier recognition. Based on those findings, imaging patterns such as the FM dyad, triad, and Yunshan sign may facilitate earlier diagnosis.^8^

Survival outcome remains suboptimal. Older age and diagnostic delay emphasize the importance of early detection. WHO-FC and peripheral edema emerged as the strongest predictors of mortality, reflecting the prognostic impact of advanced right heart dysfunction. Elevated heart rate was also independently associated with mortality, consistent with observations in pulmonary arterial hypertension.^9^ The high prevalence of suspected PH confirms that PH is a major complication of FM, underscoring the need for early referral to specialized centers. Nearly half of patients had a TB history and showed worse outcomes, suggesting TB may contribute to disease progression in a subgroup of patients. However, more than half of cases were non-TB related, indicating heterogeneous etiologies.

Limitations include the retrospective design, missing data, lack of environmental and lifestyle variables, and potential follow-up bias. Generalizability is limited to Chinese populations. Prospective and multiethnic studies are needed to validate these findings and improve prognostic models.

## Conclusions

FM is associated with delay diagnosis, high prevalence of PH, and suboptimal long-term survival. Male sex, older age, diagnostic delay, higher heart rate, peripheral edema, and worse WHO-FC independently predict mortality. TB-associated FM shows poorer prognosis. Improved awareness and earlier detection are essential to improve outcomes.

## Funding Support and Author Disclosures

This work was supported by the National Natural Science Foundation of China granted to Dr Cao (82070052). The authors have reported that they have no relationships relevant to the contents of this paper to disclose.
